# Proteins from *Lignosus tigris* with selective apoptotic cytotoxicity towards MCF7 cell line and suppresses MCF7-xenograft tumor growth

**DOI:** 10.7717/peerj.9650

**Published:** 2020-08-04

**Authors:** Boon Hong Kong, Kean Hooi Teoh, Nget Hong Tan, Chon Seng Tan, Szu Ting Ng, Shin Yee Fung

**Affiliations:** 1 Medicinal Mushroom Research Group, Department of Molecular Medicine, Faculty of Medicine, University of Malaya, Kuala Lumpur, Wilayah Persekutuan, Malaysia; 2 Department of Pathology, University of Malaya, Kuala Lumpur, Malaysia; 3 Ligno Research Initiative, Ligno Biotech Sdn Bhd, Balakong Jaya, Malaysia; 4 Center for Natural Products Research and Drug Discovery, University of Malaya, Kuala Lumpur, Malaysia; 5 University Malaya Centre for Proteomics Research, Faculty of Medicine, University of Malaya, Kuala Lumpur, Malaysia

**Keywords:** *Lignosus tigris* (Polyporaceae), Proteins, Cytotoxicity, Apoptosis, In vivo antitumor

## Abstract

**Background:**

*Lignosus tigris*, a recently discovered species of the unique *Lignosus* family, has been traditionally used by the indigenous communities in Peninsular Malaysia to treat various ailments and as an alternative medicine for cancer treatment. The *L. tigris* cultivar sclerotia (Ligno TG-K) was found to contain numerous bioactive compounds with beneficial biomedicinal properties and the sclerotial extract exhibited potent antioxidant activity. However, the anticancer property of the Ligno TG-K including in vitro and in vivo antitumor effects as well as its anticancer active compounds and the mechanisms has yet to be investigated.

**Methods:**

The cytotoxicity of the Ligno TG-K against human breast (MCF7), prostate (PC3) and lung (A549) adenocarcinoma cell lines was evaluated using MTT cytotoxicity assay. The cytotoxic mechanisms of the active high molecular weight proteins (HMWp) fraction were investigated through detection of caspases activity and apoptotic-related proteins expression by Western blotting. The in vivo antitumor activity of the isolated HMWp was examined using MCF7 mouse xenograft model. Shotgun LC-MS/MS analysis was performed to identify the proteins in the HMWp.

**Results and Discussion:**

Cold water extract of the sclerotia inhibited proliferation of MCF7, A549 and PC3 cells with IC_50_ ranged from 28.9 to 95.0 µg/mL. Bioassay guided fractionation of the extract revealed that HMWp exhibited selective cytotoxicity against MCF7 cells via induction of cellular apoptosis by the activation of extrinsic and intrinsic signaling pathways. HMWp activated expression of caspase-8 and -9 enzymes, and pro-apoptotic Bax protein whilst inhibiting expression of tumor survivor protein, Bcl-2. HMWp induced tumor-cell apoptosis and suppressed growth of tumor in MCF-7 xenograft mice. Lectins, serine proteases, RNase Gf29 and a 230NA deoxyribonuclease are the major cytotoxic proteins that accounted for 55.93% of the HMWp.

**Conclusion:**

The findings from this study provided scientific evidences to support the traditional use of the *L. tigris* sclerotia for treatment of breast cancer. Several cytotoxic proteins with high abundance have been identified in the HMWp of the sclerotial extract and these proteins have potential to be developed into new anticancer agents or as adjunct cancer therapy.

## Introduction

Cancer is one of the leading causes of morbidity and mortality worldwide. Based on world cancer statistics-GLOBOCAN 2018, an estimate of 18 million new cases and 9.5 million cancer deaths occurred globally in 2018 (World Health Organization, 2018). Lung and breast cancers (>2 million cases) are the most common form of cancer with the highest mortality rate causing 25% of total cancer deaths (World Health Organization, 2018). One of the reasons for the high mortality rate in cancer patients is the ineffectiveness of current chemotherapeutic drugs that lack target specificity, typically causing systemic toxicity and various side effects including fatigue, hair loss, infection and organ dysfunction, which leads to poor quality of life in patients. Therefore, there is a continual need to search for novel, effective and more specific therapeutic agents.

Mushrooms are popular natural products used not only to enhance the immune system but as a complementary and alternative medicine in cancer therapy due to its indicative anticancer effects (Sullivan, Smith & Rowan, 2006). The *Lignosus* spp., fungi of the Polyporaceae family, which consists of eight different species (*L. dimiticus, L. ekombitii, L. goetzii, L. sacer, L. rhinocerus, L. hainanensis, L. tigris and L. cameronensis*) have been known for its medicinal and functional properties for many centuries (Lau et al., 2015). The anticancer properties of the *L. rhinocerus* have been extensively investigated. Lai, Wong & Cheung (2008) reported the antiproliferative activity of polysaccharide-protein complexes from *L. rhinocerus* on different leukemic cells. The cold water extract prepared from sclerotium of *L. rhinocerus* has also been reported to exhibit cytotoxicity against of a panel of human adenocarcinoma and carcinoma cell lines including breast, lung, leukemic, liver, colorectal, prostate, oral squamous and nasopharyngeal cancers, and proteins and polysaccharide-protein complexes have been hypothesized as the active cytotoxic components (Lee et al., 2012; Lau et al., 2013). A subtilisin-like serine protease fraction purified from *L. rhinocerus* sclerotia was also shown to exhibit cytotoxicity against MCF7 cells via induction of apoptosis (Yap et al., 2018).

In 2013, a distinct, new species of *Lignosus* mushroom was discovered from the tropical forest in the state of Pahang, Malaysia (Tan, Ng & Tan, 2013). This mushroom had not been formerly distinguished from *L. rhinocerus* due to its similarities in gross morphologies and has often been mistakenly recognized as *L. rhinocerus*. The identity of the new species has since been verified using DNA barcode marker targeting the internal transcribed spacer (ITS) region and was named *Lignosus tigris Chon S. Tan* (Tan, Ng & Tan, 2013; Yap et al., 2014c). The wild type of *L. tigris* sclerotia is reported to contain good nutritive value and the sclerotial extracts possess potent antioxidant capacity (Yap et al., 2014a). The *L. tigris* mushroom has recently been successfully cultivated and we have evaluated the toxicity profile of the cultivated *L. tigris* sclerotium (also known as Ligno TG-K). The Ligno TG-K was shown to be safe for consumption and contained bioactive components that exhibit immunomodulatory and anticancer properties (Kong et al., 2016a). The local Malaysian natives have claimed the medicinal benefits from *Lignosus* sclerotia consumption in treating various illnesses including breast cancer. To date, there has been no study conducted to evaluate the anticancer properties of the *L. tigris* sclerotia. In this study, we aim to investigate the in vitro cytotoxicity and in vivo antitumor activity of Ligno TG-K as well as to look into the protein component(s) responsible for these bioactivities.

## Materials & Methods

### Mushroom sample

*L. tigris Chon S. Tan* mushroom sclerotium was collected from a tropical forest in Lata Iskandar (4°17.46′N101°34.41′E), Pahang, Malaysia (Tan, Ng & Tan, 2013). The sclerotium specimen was deposited (K(M) 177826) at Mycological Herbarium, Royal Botanic Gardens, Kew (Richmond, London, UK). *L. tigris* sclerotium (Ligno TG-K) was cultivated by Ligno Biotech. Sdn. Bhd. (Selangor, Malaysia) and each batch of Ligno TG-K was verified by DNA barcode marker targeting the internal transcribed spacer (ITS) region (Kong et al., 2016b; Yap et al., 2014c). Freeze dried Ligno TG-K powder used in this study was provided by Ligno Biotech.

### Cold water extraction, fractionation and determination of total protein, carbohydrates, glucan type and terpenoids

Cold water extraction was performed by dissolving the mushroom sclerotial powder in distilled water, in a ratio of 1:20 (g/mL) and stirred continuously at 4 °C for 24 h. The extract mixture was centrifuged at 8,000 g for 30 min and the supernatant was filtered through Whatman^®^ Grade 1 filter paper prior to freeze-drying. The Ligno TG-K cold water extract (CWE) was fractionated by Sephadex G-50 (fine) gel filtration chromatography with 0.05 M ammonium acetate as eluent. Elution was carried out by gravity with a flow rate of two mL/min. Three pooled fractions of high (HMW), medium (MMW) and low (LMW) molecular weights were collected based on the distribution of the protein and carbohydrate in each fraction. The initial protein and carbohydrate contents in each fraction were estimated by Bradford (Bradford, 1976) and phenol sulfuric acid assays (Dubois et al., 1956), respectively. Collected pooled fractions were freeze dried and kept at −20 °C for further analysis. Total protein and carbohydrate contents of the pooled fractions were determined using 2D Quant Kit (GE Healthcare) and phenol sulfuric acid assay (Dubois et al., 1956), respectively. The glucan content in the fractions were determined using Megazyme Mushroom and Yeast Beta-Glucan Assay Kit (Megazyme International Ireland Ltd, Wicklow, UK) (Kong et al., 2016a). Total terpenoid content was estimated by using linalool as the standard as described previously (Ghorai et al., 2012; Kong et al., 2016a).

### Ammonium sulfate precipitation of CWE-HMW proteins

Proteins in the CWE-HMW fraction were precipitated by using 100% saturation ammonium sulfate (stirring at 4 °C for 1 h). Precipitated proteins were then collected by centrifugation followed by desalting using Vivaspin^®^ Centrifugal Concentrator 15R (MWCO-5 kDa) (Sartorius Stedim Biotech, Germany). The non-protein component (supernatant) was recovered using Sephadex G-25 (fine) gel filtration chromatography by desalting.

### Cytotoxicity assay

Human breast (MCF7), lung (A549) and prostate (PC3) cancer cell lines and non-tumorigenic breast (184B5) cell line were purchased from ATCC^®^ (Virginia, USA). MCF7, PC3 and A549 cells were maintained in Roswell Park Memorial Institute 1640 medium (RPMI-1640, Biowest, France) supplemented with 10% fetal bovine serum (FBS). 184B5 cells were maintained in MEBM medium supplemented with MEGM bullet kit (Lonza, Basel, Switzerland) and 10% FBS. Cells were cultured in a humidified carbon dioxide (5%) incubator with temperature set at 37 °C.

The cytotoxic effect of extract and fractions was examined using the MTT assay as described in Mosmann (1983) with some modifications. Cells with optimal density was seeded into a 96-well microplate and incubated overnight prior to treatment with various concentrations of samples for 72 h. At the end of the incubation time, 20 µL of MTT solution (5 mg/mL) was added and cells were incubated in the dark at 37 °C for 4 h. All the solution was removed by aspiration and 200 µL of DMSO was added to dissolve the purple formazan crystals. Absorbance readings were measured using a microplate reader at 570 nm. A plot showing percentage of cell viability against the tested sample concentration was used to determine the half-maximal inhibitory concentration (IC_50_).

### Caspases activity

Caspases activation was evaluated using Caspase-Glo^®^ kits (Promega, USA), according to the manufacturer’s protocol. Briefly, MCF7 cells were seeded into 96-well white plate and treated with HMWp (at IC_50_ value) for 12 h, 24 h, 48 h and 72 h. After treatment, Caspase-Glo^®^ reagent was added in 1:1 ratio into each well and incubated at room temperature for 1 h. The luminescent signal was measured using monochromator microplate reader, Tecan^®^ Infinite M1000 Pro (Tecan Austria GmbH).

### Western blot analysis

MCF7 cells were treated with HMWp at IC_50_ value for 24 h, 48 h and 72 h. Total protein was extracted from the treated cells by using RIPA lysis buffer as described by Yap et al. (2018). Thirty micrograms of protein were separated on 12.5% SDS-PAGE and transferred onto polyvinylidene difluoride membrane. The membrane was blocked with 5% w/v skim milk in TBST (1X TBS, 0.1% Tween-20) for 1 h, and incubated at 4 °C with the corresponding primary antibodies: a β-actin rabbit monoclonal antibody (1:1000), a Bax rabbit monoclonal antibody (1:1000), a Bcl-2 rabbit monoclonal antibody (1:200) and a BID polyclonal antibody (1:1000) (Cell Signaling Technology, Inc., MA, USA), prepared in 5% w/v BSA in TBST. After incubation, membranes were washed with TBST and followed by incubation with HRP-anti rabbit IgG (diluted 1:2000 in 5% skim milk, TBST solution) for 1 h at room temperature. HRP enzyme activity was detected using enhanced luminol-based chemiluminescent substrate (Pierce™ ECL Western Blotting Substrate, Thermo Scientific, USA) and chemiluminescent signal was captured using a CCD-based imaging system (BioSpectrum^^®^^ Imaging System, UVP, USA). Quantitative analysis of the signal intensity was performed using ImageJ software (Schneider, Rasb & Eliceiri, 2012) and the protein expression level was determined by normalization to the internal control β-actin signal intensity.

### In vivo MCF7 tumor xenograft mouse model

#### Animal and experimental procedures

Animal experimental protocols were approved (October 29, 2013) by the Institutional Animal Care and Use Committee, University of Malaya (UM IACUC - Ethics reference no. 2013-09-17/MOL/R/TNH). Female athymic NCr nude mice (4 weeks old) were purchased from InVivos, Singapore. The mice were housed under specific pathogen-free environment with temperature set between 22 ± 2 °C under 12 h light/dark cycle and relative humidity of 60 ± 10%. All the mice were given an unlimited supply of sterile food (Altromin #1324 Rodent Maintenance Diet, Denmark) and water. The mice were quarantined and acclimatized to laboratory conditions for 14 days prior to experimental procedures. 17β-estradiol (E_2_) silastic capsules containing 1:31 of E_2_ and cholesterol in a total of 3 mg (Ju et al., 2006) were implanted subcutaneously in dorsal flank of the mice (*n* = 12). Four days after the implantation, 200 µL of an equal mixture of MCF7 cells (1 × 10^6^ cells) and Matrigel™ was injected subcutaneously into the mammary fat pad of the mice. Tumor growth was monitored over time using an electronic caliper. Tumor volume (mm^3^) was determined using the following formula (Faustino-Rocha et al., 2013):

\begin{eqnarray*}\text{Tumor volume} ({\text{mm}}^{\text{3}})=\text{Length (mm)}\times [\text{Width (mm)}]^{2}/2. \end{eqnarray*}

Mice were randomly assigned into PBS control group (*n* = 6) and HMWp treatment group (*n* = 6) when the tumor volume reached 100–150 mm^3^. Treatment group received intraperitoneal (*i.p.*) administration of 5 µg/g of body weight/200 µL of HMWp on alternate days over a period of 21 days. Mice were observed for behavioral changes at 2 h after dosing and at 24 h for mortality and signs of toxicity. Throughout the study, animals that showed severe pain or signs of enduring severe distress, having a tumor burden greater than 10% body weight, bearing a tumor that exceeds 15 mm in any one dimension, having tumors that had ulcerated/necrotized/infected and tumors that interfered with eating or impaired ambulation were included as criteria of humane endpoints. The tumor volume and body weight of individual mice were monitored and recorded weekly over the 21-day treatment period. At day 22, all the mice from both treated and control groups were euthanized using carbon dioxide.

### Histopathological analysis

At day 22, mice were euthanized and the vital organs (lung, liver, heart, spleen, kidneys) and solid tumor tissues were harvested and fixed in 10% buffered formalin for 48 h for microscopic examination. The fixed tissues were dehydrated by serial ethanol solution, cleared with xylene and subsequently paraffin embedded. The vital organ tissue sections were stained with hematoxylin and eosin and examined under light microscope.

### Detection of apoptotic cells by TUNEL

Apoptotic cells in the tumor tissues were detected using TUNEL method by Apoptag^®^ Plus Peroxidase *In Situ* Apoptosis Detection Kit (Millipore, MA, USA). TUNEL stained apoptotic cells in the tumor tissue sections were observed under light microscope and 5 random fields of view per section were captured at 100 × magnification. The percentage of apoptotic cells was calculated as the number of apoptotic cells over the total number of cells in each field using the ImageJ software (Schneider, Rasb & Eliceiri, 2012).

### 1D SDS-PAGE-nano-ESI-LC-MS/MS analysis

HMWp (30 µg) was subjected to SDS-PAGE and proteins bands separated on the gel were excised into 6 sections S1-S6. In-gel digestion of the proteins was carried out as described by Yap et al. (2015b). Briefly, destained protein sections were subjected to reduction with 10 mM dithiothreitol and alkylation with 55 mM iodoacetamide prior to digestion with trypsin (Pierce™ Trypsin Protease, Thermo Scientific, USA). The tryptic digested peptides were desalted and the sample (in 0.1% formic acid) was subjected to nano-ESI-LC-MS/MS using Agilent 1260 HPLC-Chip/MS Interface, coupled with Agilent 6550 Accurate-Mass Q-TOF LC/MS (Agilent Technologies, California, USA). Two microliters of sample were injected into the microfluidic nanospray chip containing a 160-nl enrichment column packed with C18 (300 Å) at 4 µL/min. Analytical separation of the peptides was completed through the pre-column in-line with a 75 µm × 150 mm analytical column at 0.4 µL/min in a linear gradient from Solution A (0.1% formic acid in water) to 95% Solution B (100% acetonitrile, 0.1% formic acid in water) in 47 min including post-run of 8 min. MS/MS data acquisition parameters were as previously described in Yap et al. (2015b) with minor adjustments. For subsequent MS (rate: 20 spectra/s, time: 50 ms/spectrum) and MS/MS (rate: 8 spectra/s, time: 125 ms/spectrum) analyses, spectra were acquired in a MSMS mode with scan range from 200 to 3,000 m/z and 50 to 3,200 m/z, respectively. Capillary and fragmentor voltage were 1,800 V and 175 V, respectively, with drying gas flow rate of 11.0 L/min at 290 ° C. MS/MS spectra were searched using Agilent Spectrum Mill MS Proteomics Workbench software packages (http://spectrummill.mit.edu/) against *L. rhinocerus* TM02 genome database (Yap et al., 2014b). The spectrum mill settings were set as follows: MH+ scan range from 100 to 3,200 Da, complete carbamidomethylation of cysteines, filter protein FDR >0%, filter peptides FDR at <1.0% and filter protein score by 20.0 and above. Only results with “Distinct Peptide” identification of 2 and above are considered significant.

Relative protein content in terms of percentage in each excised section was determined using the following formula:

\begin{eqnarray*}\text{Percentage of protein in each gel section}=x/\Sigma x\times \text{relative intensity (%)} \end{eqnarray*}

where, x = mean peptide spectral intensity of a protein

Σx = total mean peptide spectral intensity of proteins.

Relative intensity (%) of each gel section in a protein lane was estimated by densitometry using Thermo Scientific™ Pierce™ myImage Analysis™ Software (USA).

### Statistical analyses

All data were expressed as mean ± standard deviation (SD) unless stated otherwise. Data of the chemical compositions in Ligno TG-K CWE fractions were analyzed using one-way analysis of variance (ANOVA) followed by least significant difference (LSD) post hoc test (IBM SPSS Statistics 22) to evaluate the differences between the mean values in the experiment groups. Statistical differences between the mean of untreated/control and treated groups in cytotoxicity, apoptosis and antitumor studies were analyzed using independent samples *t*-test. All differences were considered statistically significant at *p* < 0.05.

## Results

### Chemical compositions of Ligno TG-K cold water extract and its fractions

Cold water extract (CWE) of the Ligno TG-K contains 41% and 7% carbohydrates and proteins, respectively. The CWE was fractionated into HMW, MMW and LMW fractions (Fig. S1). The LMW fraction has the highest yield (79.2%) amongst the CWE fractionated materials, followed by HMW (15.5%) and MMW (5.3%) fractions. The HMW fraction contained the highest protein content whilst MMW fraction contains the highest carbohydrate content (Table 1). LMW consists of mainly carbohydrates with very low amounts of proteins (<1%). Glucan content determination of the fractions revealed that alpha glucan (+ oligomers etc.) was the major glucan component in all the fractions tested (Table 1). Both MMW and LMW contain comparable β-glucan content. The terpenoid level in LMW was found to be at least 5 and 3 times higher than the HMW and MMW, respectively (Table 1).

### Cytotoxic activity

The CWE exhibited the most potent cytotoxicity on MCF7 cells with an IC_50_ of 28.93 ± 7.74 µg/mL, which is approximately 6-fold lower than the non-tumorigenic breast 184B5 cells, (IC_50_ of 183.67 ± 34.70 µg/mL). Although CWE showed significant cytotoxicity on lung cancer A549 (IC_50_ of 70.67 ± 16.17 µg/mL) and prostate cancer PC3 (IC_50_ of 95.00 ± 7.07 µg/mL) cells, it was also cytotoxic to the non-tumorigenic lung NL20 (IC_50_ value of 71.83 ± 5.13 µg/mL) and prostate RWPE1 (IC_50_ of 1.63 ± 0.04 µg/mL) cells. Further fractionation of the CWE showed that the HMW has strongest cytotoxicity against MCF7 cells at which the IC_50_(4.23 ± 0.08 µg/mL) were at least 8-fold lower than MMW (IC_50_ of 34.75 ± 1.78 µg/mL). LMW did not exhibit significant cytotoxicity (IC_50_>500 µg/mL) on MCF7 cells. The HMW showed less cytotoxicity to 184B5 cells (IC_50_ of 13.10 ± 1.98 µg/mL). The protein (HMWp) and non-protein (HMWnp) components of HMW was subsequently separated using ammonium sulfate precipitation. The HMWp showed strong cytotoxicity against MCF7 cells with an IC_50_ of 1.17 ± 0.47 µg/mL and was about 3-fold lower than the 184B5 cells (IC_50_ of 3.43 ± 0.60 µg/mL). No significant cytotoxic activity was observed in HMWnp on the MCF7 cells (IC_50_ >500 µg/mL).

**Table 1:** Chemical compositions of the Sephadex G-50 fractions of CWE.

Fraction	Carbohydrate (%)	Protein (%)	Glucan (% DW of carbohydrate content)			Total terpenoid
			α-glucan (+ oligomers etc.)	β-glucan	Total glucan (+ oligomers etc.)	mg LE/g fraction
HMW	27.8	18.0	40.44 ± 0.33^a^	0.04 ± 0.82^a^	40.48 ± 0.57^a^	207.78 ± 20.94^a^
MMW	49.7	5.9	57.94 ± 0.23^b^	9.13 ± 0.77^b^	67.07 ± 0.82^b^	362.92 ± 17.18^b^
LMW	29.4	0.7	55.75 ± 1.03^c^	10.25 ± 0.68^b^	66.01 ± 0.62^b^	1216.18 ± 120.30^c^

**Notes.**

Total terpenoid content is expressed as mean ± SD (*n* = 6). All the other data are expressed as mean ± SD (*n* = 3). Different superscript letters in the same column indicate the mean values are significantly different (SPSS, one-way ANOVA and LSD post hoc test, *p* < 0.05).

Abbreviations HMW high molecular weight MMW medium molecular weight LMW low molecular weight LE linalool equivalent DW dry weight

### Effects of HMWp on caspases and apoptotic protein expression levels

**Figure 1:**
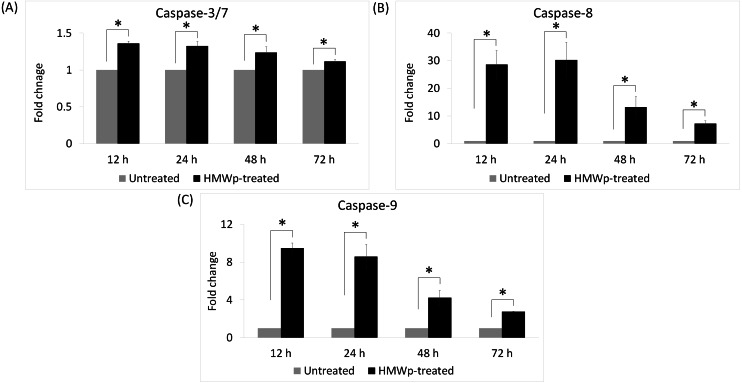
Caspase-3/7, -8 and -9 activities of the HMWp-treated MCF7 cells. (A–C) Cells were treated with 1.2 µg/mL of HMWp and caspase activities were measured at 12 h, 24 h, 48 h and 72 h. Data are expressed as mean ± standard error (S.E.M) from two independent experiments performed in triplicate.^∗^ indicates significant difference in the fold change between the treated and untreated control cells (SPSS, independent samples *t*-test, *p* < 0.05).

To investigate the involvement of caspases in HMWp-induced apoptosis, caspase-3/7, -8 and -9 activities of MCF7 cells treated with HMWp (1.2 µg/mL) for 12 h, 24 h, 48 h and 72 h were measured using the Caspase-Glo^®^ kits. As shown in Fig. 1, caspase-3/7 activities were significantly higher after treatment with HMWp. Both the caspase-8 and -9 activities peaked at 12 h and 24 h to about 30-fold and 9-fold greater than the untreated cells, respectively, and gradually decreased over the 72 h incubation period.

**Figure 2:**
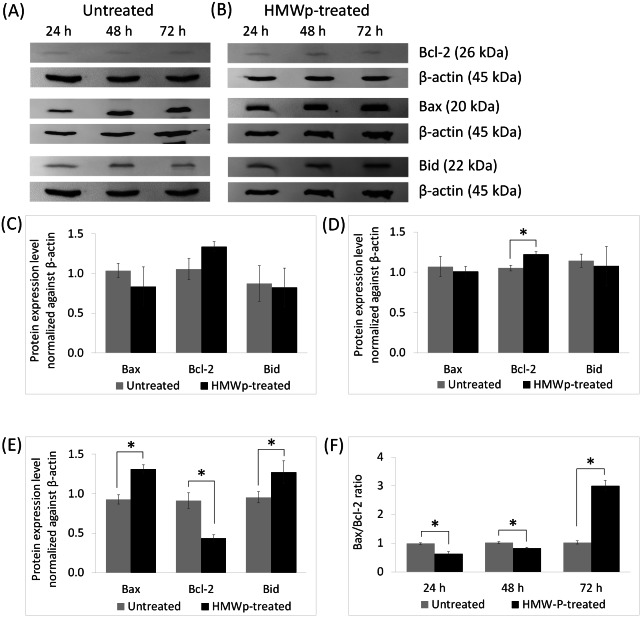
Effects of HMWp on apoptosis-related protein expression in MCF7 cells. Western blot analysis of Bcl-2 family proteins (Bcl-2, Bax, and Bid) from (A) untreated control and (B) HMWp treated MCF7 cells at 24 h, 48 h and 72 h. The untreated control and HMWp-treated samples were derived from the same experiment and that gels/blots were processed in parallel. β-actin was served as internal control. Relative protein expressions of Bcl-2, Bax and Bid in MCF7 cells at (C) 24 h, (D) 48 h and (E) 72 h, were determined by normalization to β-actin. (F) Bax/Bcl-2 ratio in untreated control and HMWp-treated MCF7 cells. Treatment with HMWp for 72 h increased the Bax/Bcl-2 ratio compared with untreated control. Data are expressed as means ± SD from three independent experiments. * indicates significant difference in expression level/fold change between the untreated control and treated cells (SPSS, independent samples *t*-test, *p* < 0.05).

Western blot analysis on the expression level of apoptotic-related proteins including Bcl-2, Bax and Bid showed HMWp suppressed the Bcl-2 protein level but increased the expression of Bax and Bid proteins in MCF7 cells (Figs. 2A, 2B and 2E). There was no cleaved Bid detected after 72 h of HMWp treatment (Figs. 2A and 2B). Exposure to HMWp at 72 h also led to an increase of Bax/Bcl-2 ratio of about 3-fold compared with the untreated cells (Fig.  2F).

### In vivo antitumor activity of HMWp

#### Suppression of tumor growth by HMWp

**Figure 3:**
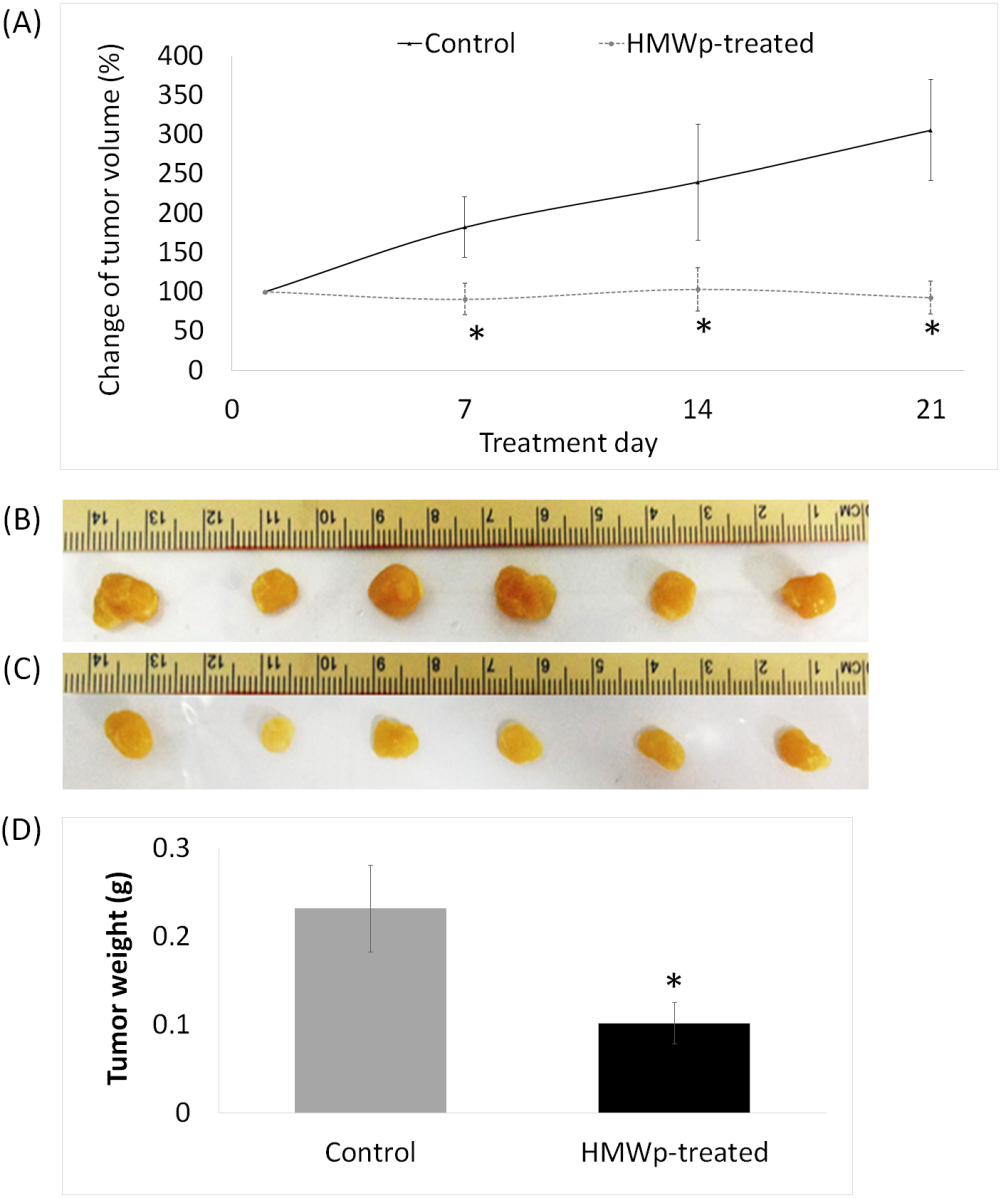
Effects of HMWp in suppression of MCF7 tumor xenografted in NCr nude mice. (A) Percentage change of the MCF7 tumor volume on the NCr nude mice treated with HMWp (*i.p.*) (5 µg/g BW/200 µL, *n* = 6) or PBS (control, *n* = 6) alternate-day for 21 days. Tumor volume was measured weekly and the percentage change of tumor volume was calculated by comparing to volume on day 1 (assigned as 100%). Tumors harvested at the end of treatment (day 22): (B) control mice treated with PBS and (C) HMWp-treated mice. (D) Average weight of tumors harvested from control and treated mice. Data are expressed as mean ± SD (*n* = 6). * indicates significant difference between the control and treated group (SPSS, independent samples *t*-test, *p* < 0.01).

A total of 11 *i.p.* treatments of HMWp (5 µg/g BW) in MCF7 tumor bearing mice over a period of 21 days had successfully suppressed the growth of the tumors. The tumor volume of the HMWp-treated mice was significantly (*p* < 0.01) smaller than the control mice (Fig. 3A). There were no significant changes in the average volume of the tumors in the HMWp-treated mice throughout the 21 days. In contrast, the tumors in the control mice grew 3 times larger than its original size. At the end of the experiment, all the control and HMWp-treated mice were sacrificed, and their tumors were excised (Figs. 3B and 3C). The tumor weight of HMWp-treated mice was significantly (*p* < 0.01) lower than the control mice (Fig. 3D).

#### HMWp induced apoptosis in solid MCF7 tumors

**Figure 4:**
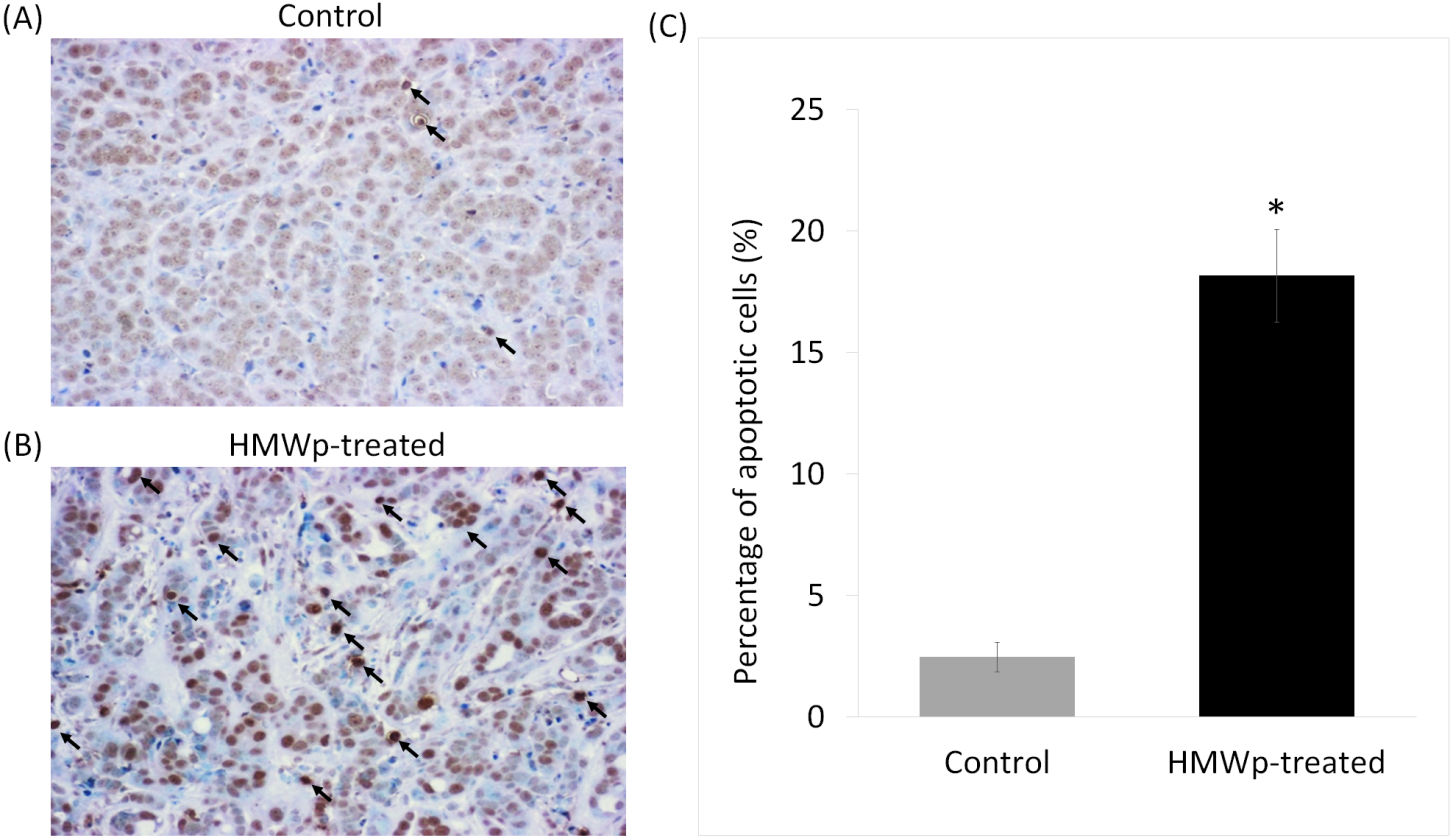
Apoptotic effect of HMWp treatment on MCF7 tumors. Tumor tissue sections from mice treated with (A) PBS (control) and (B) HMWp were stained with TUNEL (representative images at 100 × magnification). Dark arrows indicate the apoptotic cells (stained in dark brown). (C) Percentage of TUNEL-positive cells detected in tumor sections from PBS control and HMWp-treated mice. Data are expressed as mean ± SD (*n* = 6). ^∗^ indicates significant difference in the average percentage of TUNEL-positive cells in control and treatment groups (*p* < 0.05).

In tumor tissues of the treated mice, HMWp greatly increased the number of TUNEL-positive apoptotic cells (stained in dark brown) in comparison with the PBS control (Figs. 4A and 4B). Quantification using ImageJ software showed that the average percentage of TUNEL-positive cells in HMWp-treated mice (18.2%) was significantly (*p* < 0.05) higher than the control mice (2.5%) (Fig. 4C ).

#### Toxic effects of the HMWp on tumor bearing mice

**Figure 5:**
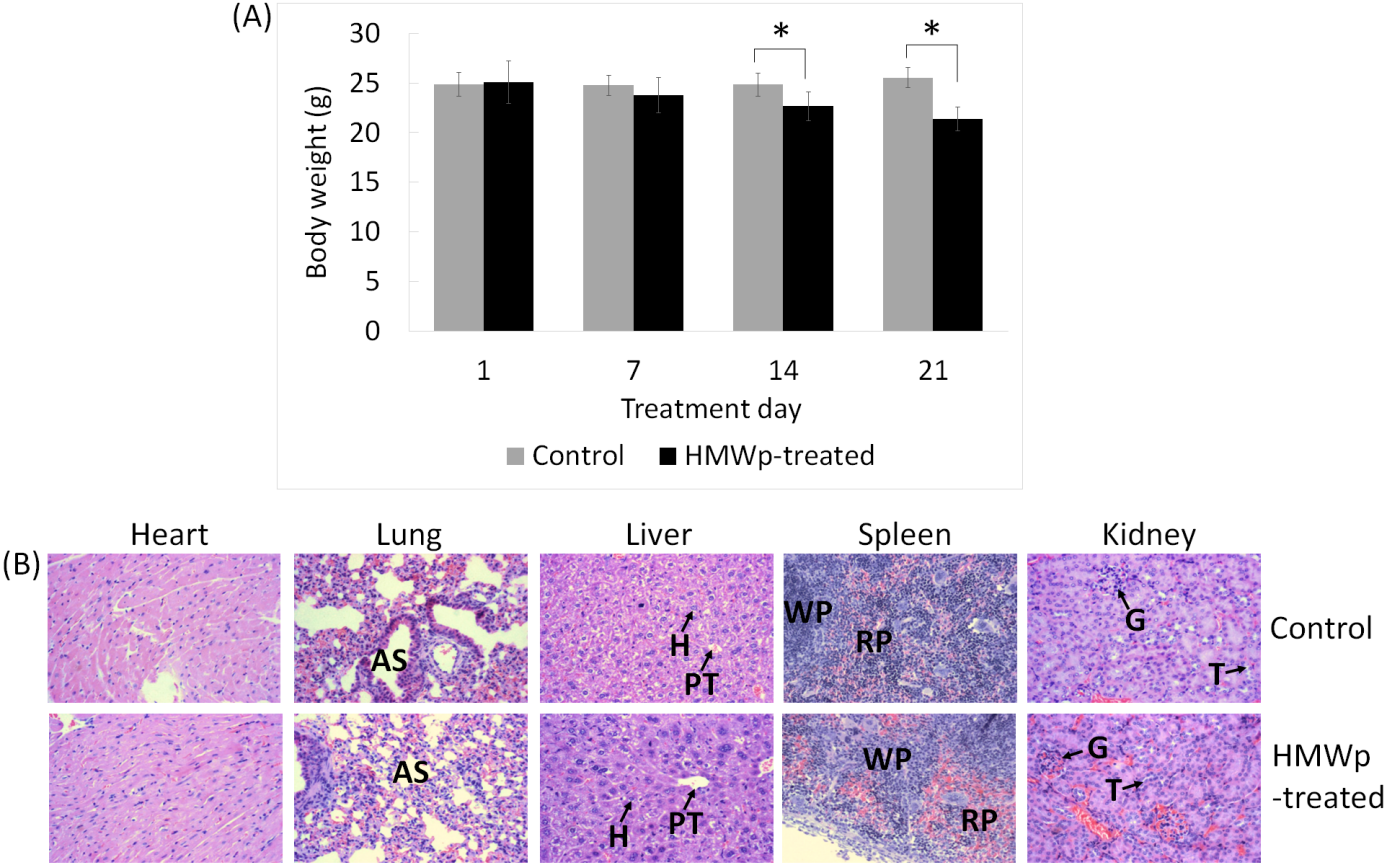
The effects of HMWp on body weight and vital organs of the mice. (A) Body weight change in control and HMWp-treated mice. Body weight is shown as mean ± SD (*n* = 6). Data were analyzed using SPSS, independent samples *t*-test.^∗^ indicates significant difference in the body weight between control and treatment groups (*p* < 0.05). (B) Histological sections of vital organs of control and HMWp-treated mice. Microscopy examination of the hematoxylin and eosin stained tissues showed no significant histopathological abnormalities in the organs (heart, lung, spleen, kidney and liver) of control and treated rats. Abbreviations: AS, alveolar space; PT, portal tract; H, hepatocyte; WP, white pulp; RP, red pulp; G, glomerulus; T, tubule. (100 × magnification).

The behavioral changes and body weight of the mice were monitored throughout the study and histopathological analysis on the vital organs (lung, liver, heart, spleen and kidneys) was performed to assess the toxic effects of the HMWp on the mice. Treatment with HMWp induced writhing reaction in the mice and the pain subsided at 1 h post injection (*i.p.*). Decreased locomotor activity among the treated mice was observed at the first 24 h. The body weight of the mice did not show significant change in the first week of treatment; however, the treated mice begun showing reduced body weight on day 14 post-treatment (*p* < 0.05) (Fig. 5A).

Histopathological analysis showed that the HMWp treatment (at a dose of 5µg/g BW, alternate day over a period of 21 days) did not cause any gross pathological lesions in the vital organs (heart, lung, liver, spleen and kidneys). The histological sections of heart, lung, liver, spleen and kidney of the control and treated mice are shown in Fig. 5B.

### Protein composition of HMWp

**Figure 6:**
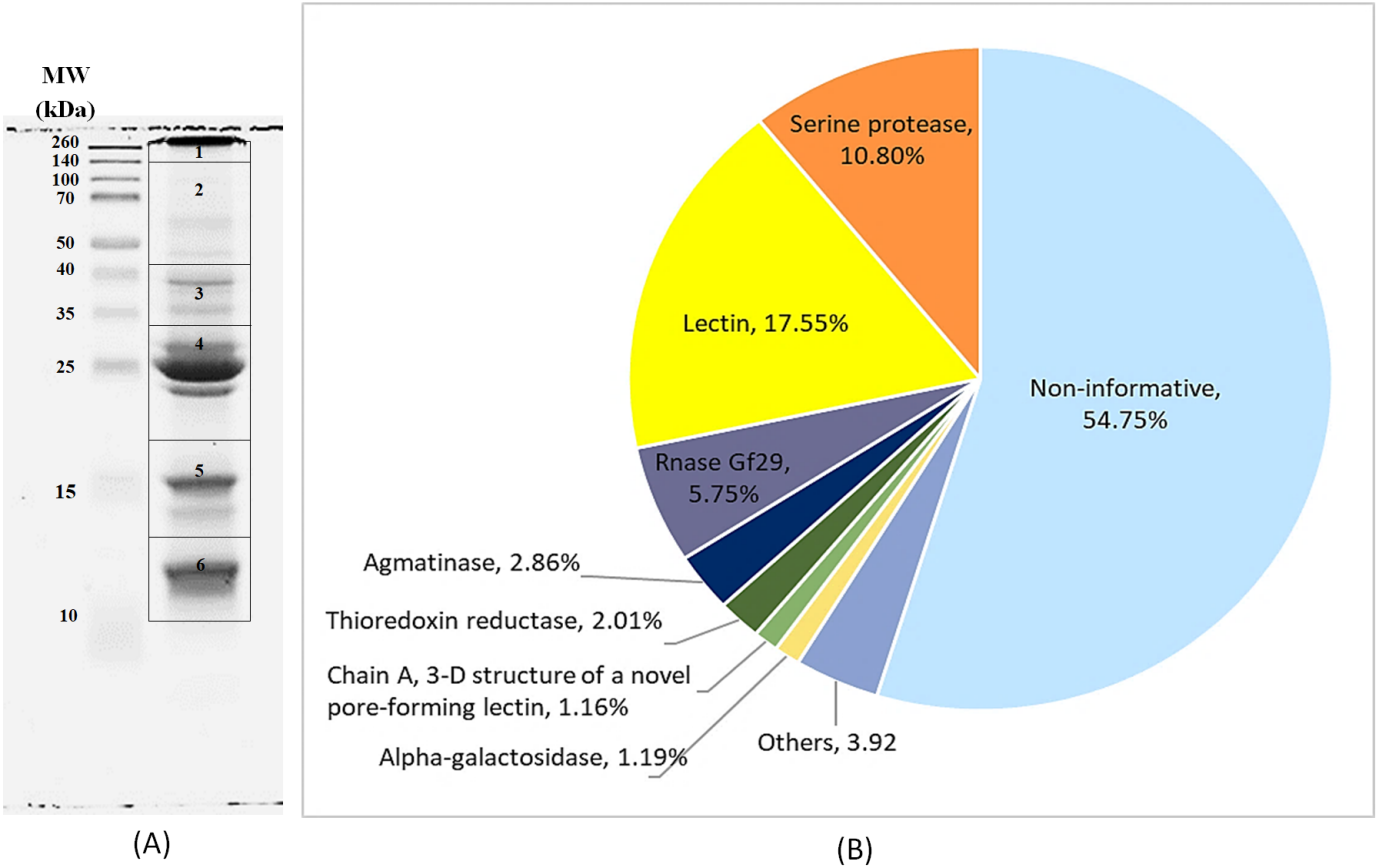
Protein composition of HMWp. (A) Protein profile of HMWp on SDS-PAGE gel (15%). The gel was cut into 6 sections (labelled 1 to 6) and digested with trypsin before LC-MS/MS analysis. (B) Percentage distribution of HMWp proteins identified by label-free shotgun proteomic (Q-TOF LC/MS) based on *L. rhinocerus* TM02 genome database. Proteins with unknown and/or uncharacterized functions (including predicted, hypothetical and not annotated) were grouped as non-informative (54.75% of the total proteins). Proteins with less than 1.0% abundance were grouped as “Others” (3.92%).

Using the 1D-SDS-PAGE coupled with shotgun LC-MS/MS proteomics analysis, a total 44 different types of proteins were identified in the HMWp. Similar proteins (protein redundancy) detected in different gel sections (Fig. 6A, Table S1), likely due to the possible protein degradation and/or fragmentation throughout the extraction, purification and separation processes were removed from the raw data using gene ID as reference. The relative percentage of each protein was calculated and summarized in Fig. 6B. The non-informative proteins which include predicted, hypothetical and not annotated proteins constitute 54.75% of the total proteins. Lectin (17.55%) was the most abundant protein identified in HMWp, followed by serine protease (10.80%) and RNase Gf29 (5.75%). Proteins with less than 1.0% abundance were grouped as “Others” (3.92%).

It was interesting to note that protein 230NA with unknown function comprises about 40% of the total non-informative protein or 21.83% of the total protein in HMWp. The 230NA was mostly detected in gel section 4 (7.61%) and 5 (7.73%) (Table S1). The protein sequence GME1230_g of the 230NA obtained from TM02 database was blasted against the NCBInr database and it matched to a hypothetical protein TRAVEDRAFT_74601 (*Trametes versicolor* FP-101664 SS1) (accession no.: XP_008038372.1) with 47% identity and contains the conserved domain of DNase_NucA_NucB (accession: Cdd:pfam14040) which acts as a deoxyribonuclease.

## Discussion

Medicinal fungi are a rich source of biologically active compound with potent pharmacological activities and its popularity as a complementary and alternative medicine has been increasing in cancer therapies of late. In this study, we demonstrated that the cold water extract from *L. tigris* mushroom sclerotia (Ligno TG-K) exerted potent cytotoxic effect on MCF7 breast cancer cells. The CWE exhibited less cytotoxicity to non-tumorigenic breast 184B5 cells indicating that the extract is selectively cytotoxic. This result provided scientific evidence to support the traditional usage of this mushroom for treatment of breast conditions such as swollen breast and cancer (Ismail, 2010; Mohammad, Milow & Ong, 2012). The CWE also showed significant cytotoxicity against lung and prostate cancer cells; however, it was also cytotoxic to the respective non-tumorigenic cells, indicating lack of the cytotoxic selectivity against these cancer cells.

Previous studies from our group reported that the cold water extract from the sclerotia of *L. rhinocerus* TM02 exhibited selective antiproliferative activity against MCF7 cells (Lee et al., 2012; Yap et al., 2013). In this study, we found that Ligno TG-K CWE has stronger cytotoxic effect (IC_50_ at least 3-fold lower) than TM02 CWE. The cytotoxicity of the Ligno TG-K CWE is attributed to high molecular weight components. The HMW fraction isolated from Ligno TG-K CWE exerted selective cytotoxicity against MCF7 cells with an IC_50_ of at least 3-fold lower compared to 184B5 cells. The HMW of Ligno TG-K CWE also showed stronger cytotoxic effect against MCF7 cells (4.23 µg/mL) compared to that of TM02 (70.0–106.7 µg/mL) (Lee et al., 2012; Yap et al., 2015b). HMW was found to contain mostly protein and a low amount of beta-glucan and terpenoids. Beta-glucan and terpenoids have been reported to possess potent anticancer activity (Duru & Çayan, 2015; Zhang et al., 2006). Although beta-glucan and terpenoids were found abundantly in MMW and LMW, yet the MMW and LMW were less cytotoxic and not active against MCF7 cells, respectively. Thus, we postulated that proteins which were found abundantly in the HMW could be responsible for the cytotoxicity observed.

To further explore the mechanism of cytotoxicity of the proteins in HMW, the protein components were isolated by precipitation using 100% saturated ammonium sulfate. HMWp was found to effectively inhibit the proliferation of MCF7 cells and was selective against MCF7 cells as shown by its 3-fold lower IC_50_ compared to 184B5 cells. In addition, its cytotoxic selectivity was greater than doxorubicin, a standard chemotherapy drug used for breast cancer. Doxorubicin was more cytotoxic to 184B5 cells (IC_50_ of 0.12 ± 0.04 µg/mL) compared to MCF7 cells (IC_50_ of 0.76 ± 0.19 µg/mL) (determined using the same protocol in this study). The HMWp has the potential to be considered as a therapeutic cytotoxic agent against MCF7 cells as it fits into the criteria established by National Cancer Institute (USA), where crude extracts and pure compounds with IC_50_ of less than 20 µg/mL and 4 µg/mL at 72 h post incubation, respectively, are regarded as potentially active cytotoxic agents (Boik, 2001).

Further analysis demonstrated that the cytotoxicity of HMWp was associated with induction of apoptosis in MCF7 cells. Cell morphology changes including cell shrinkage, membrane blebbing and apoptotic bodies indicated the occurrence of apoptotic events in MCF7 cells upon treatment with HMWp (Fig. S2). This is supported by the elevation of caspase-3/7 activity in the cells treated with HMWp. The ability of HMWp to induce apoptosis in MCF7 cells is hypothesized to be mediated by caspase activation including caspase-8 and -9. Experimental results showed that the activities of caspase-8 and -9 were up-regulated following treatment with HMWp, suggesting that induction of apoptosis is mediated via the extrinsic and intrinsic apoptotic pathways. Despite the absence of caspase-3 in MCF7 cells, both the extrinsic and intrinsic pathways eventually activated caspase-7 and led to subsequent cell death.

The Bcl-2 family proteins play a key role in activation of intrinsic (mitochondrial mediated) apoptotic pathway and are divided into three subclasses including pro-apoptotic BH3-only proteins, anti-apoptotic Bcl-2-like proteins and the effector proteins Bax and Bak (Martinou & Youle, 2011). In this study, data obtained from Western blot analysis showed the HMWp induced apoptosis was accompanied by decreased expression level of anti-apoptotic Bcl-2 and increased expression levels of effector protein Bax and pro-apoptotic Bid, without the presence of truncated Bid (tBid). Elevation of Bax/Bcl-2 ratio proved that HMWp treatment increased the susceptibility of MCF7 cells to undergo apoptosis. Bax presents in excessive levels trigger the permeabilization of outer mitochondrial membrane and releases cytochrome c which in turn activates the caspase cascade resulting in apoptosis (Tait & Green, 2010; Youle & Strasser, 2008). However, the mechanism of Bax activation in regulating the permeabilization of outer mitochondrial membrane in MCF7 cells following treatment with HMWp remains unknown and requires more investigations. The absence of the tBid suggests that there is no crosstalk between the extrinsic and intrinsic pathways. Our results suggest that HMWp activates extrinsic and intrinsic apoptotic signaling pathways independently to induce apoptosis in MCF7 cells.

In vivo studies are crucial to the drug discovery and development of novel cancer therapies. In vivo animal models allow preclinical testing of potential anticancer agents and provide useful information on toxicological and therapeutic effects, as well as pharmacokinetic properties of the drug candidates, prior to clinical trials to ensure the efficacy and safety of the drugs. Our in vivo antitumor study using MCF7 tumor xenograft model demonstrated that HMWp is effective in suppressing the growth of solid breast tumors. The HMWp induced DNA damage and apoptotic cell death in tumor cells and subsequently reducing the tumor mass in the treated mice. The dosage of HMWp (5 µg/g BW) administered to the animals was decided based on the estimation of the amount of drug that could be delivered to the tumor sites. By assuming the extracellular fluid (ECF) volume of a mouse to be ∼20% of body weight (Chapman et al., 2010), the injected dose of 5 µg/g BW is estimated to deliver about 25 µg/mL of HMWp into the ECF (assuming complete absorption) of a mouse weighing 20 g. This selected dosage of HMWp (5 µg/g BW or 25 µg/mL ECF) is approximately 20 times higher than the IC_50_ (1.17 µg/mL) of MCF7 cells in vitro. Nonetheless, in the in vitro cytotoxicity assay, HMWp was administered directly onto the monolayer MCF7 cells having direct contact with the cells. In this in vivo antitumor study, the quantity of HMWp that tumor cells are exposed to is likely to be less than amount administered (*i.p.* at 25 µg/mL ECF) as not all the administered amount of HMWp can be absorbed and delivered to the target tumor site, considering the characteristics of the solid tumor structure which includes cells distant from blood vessels, high interstitial fluid pressure and extracellular matrix (Minchinton & Tannock, 2006; Sriraman, Aryasomayajula & Torchilin, 2014) which will restrict the penetration of HMWp into the tumor cells. Hence, the amount of HMWp that the tumor cells are exposed to is far less than 25 µg/mL. In addition, this dosage was not toxic to the mice as no adverse effects was observed in the vital organs. Observation of the mice did, however revealed possible momentary pain in the treated mice. Acidic condition of HMWp due to high composition of acidic amino acids (glutamate and aspartate) in Ligno TG-K protein (Kong et al., 2016a) or presence of irritant compounds in HMWp could have induced abdominal discomfort in the mice. This condition was followed by a decrease in locomotor activity and thus reduction in food intake which subsequently led to weight loss in the mice. This is the first study demonstrating the antitumor effects of proteins isolated from a newly discovered species of Tiger Milk mushroom, *L. tigris* and warrants more investigations, including using a lower dosage and longer treatment duration.

Our previous studies have reported that the TM02 sclerotium contains lectins, serine proteases and immunomodulatory proteins which possess bio-pharmacological activities including antiproliferative, antitumor and immunomodulatory (Yap et al., 2015a; Yap et al., 2015b). Our current study shows that Ligno TG-K has a distinct cytotoxic and antitumor potency on breast cancer cells compared to *L. rhinocerus* TM02. Therefore, it is of interest to identify the protein components in HMWp. Using 1D-SDS-PAGE coupled with shotgun LC-MS/MS proteomics analysis, lectins and serine proteases are the two most abundant proteins detected in HMWp. Mushroom lectins and serine proteases have been reported to exert antiproliferative, antitumor, immunomodulatory, mitogenic and HIV-1 reverse transcriptase inhibitory effects (Ditamo et al., 2016; Li et al., 2008; Majumder, Banik & Khowala, 2016; Park et al., 2009; Sun et al., 2011). Study by *Yap et al. (2015b)* also demonstrated a partially purified serine protease protein fraction (designated as F5) isolated from *L. rhinocerus* TM02 sclerotium exhibited potent cytotoxic selectivity toward MCF7 cells. Our results showed 74.6% of the serine proteases in HMWp is encoded by the same gene as F5 serine proteases (97.3% encoded by GME4347_g).

T2 family ribonucleases play a role in a variety of biological functions including degradation of extra- or intracellular RNAs and acting as cytotoxic agents and immunomodulatory (Luhtala & Parker, 2010). ACTIBIND, a fungal T2-RNase isolated from *Aspergillus niger*, has been reported to exert active antiangiogenic and anticarcinogenic activities (Roiz et al., 2006). ACTIBIND inhibited the growth of HT-29 colon tumor xenograft and exerted preventive and therapeutic effects on dimethylhydrazine-induced colonic tumor in rats (Roiz et al., 2006). The RNase Gf29 belonging to RNAse T2 family was found in moderate amount in HMWp and it has not been reported in *L. rhinocerus* TM02.

To the best of our knowledge, this is the first study reporting a deoxyribonuclease-like protein in mushrooms, specifically Tiger Milk mushroom. This 230NA deoxyribonuclease-like protein encoded by GME1230_g contains a conserved domain of DNase_NucA_NucB is highly abundance in Ligno TG-K HMWp. The current knowledge on the function of this protein is scarce but the presence of the DNase_NucA_NucB domain suggests that it might act as a deoxyribonuclease. This is also supported by recent studies where fungal antitumor proteins with deoxyribonuclease activity were reported. Antitumor proteins from *Agrocybe aegerita, Pholiota nameko* and *Ramaria botrytis* mushrooms which possessed deoxyribonuclease activity have been reported to exhibit potent antiproliferative activity and apoptosis-inducing effect on several human cancer cells such as neuroblastoma (SH-SY5Y), HeLa, breast adenocarcinoma (MCF7) and lung adenocarcinoma (A549) cells (Chen et al., 2012; Zhang et al., 2014a; Zhang et al., 2014b; Zhou et al., 2017). Therefore, it is likely that the 230NA protein may have similar cytotoxic properties as with the other reported deoxyribonucleases which may also play a role in cytotoxicity of HMWp. Our data suggests that the lectins, serine proteases, RNase Gf29 and the novel 230NA deoxyribonuclease that constituting 55.93% of the HMWp, may contribute to the cytotoxicity of HMWp. Furthermore, Trejo-Becerril et al. (2016) has demonstrated a combination of proteases and DNaseI inhibited the growth of tumors in colon tumor-bearing mice. Thus, the high content of serine protease and deoxyribonuclease in HMWp could have contributed to the major antitumor activity in this study. Though other proteins which were found in low amounts in HMWp such as agmatinase, thioredoxin reductase, alpha-galactosidase and chain A of a novel pore-forming lectin have not been reported to exhibit antiproliferative activity, these proteins remain as bioactive proteins of interest for future investigations of this species.

## Conclusions

The findings from this study provided scientific evidences to support the traditional use of the *L. tigris* sclerotia for treatment of breast cancer. We demonstrated that a high molecular weight protein fraction isolated from Ligno TG-K selectively inhibited the growth of MCF7 cells through induction of apoptosis mediated by a mechanism involved both extrinsic and intrinsic pathways. This pioneering study has also shown the HMWp induced tumor-cell apoptosis in MCF7 solid tumor and suppressed tumor growth without causing significant toxicity to mice. Serine protease, lectin, RNase Gf29 and a novel deoxyribonuclease are the major cytotoxic proteins in the HMWp. The antitumor properties of HMWp support that HMWp has the potential to be developed into new anticancer agents or be used as adjunct therapy for breast cancer. Data from this study provide a new insight into the antitumor activity of Ligno TG-K and on-going studies are focused on the purification and characterization of the identified cytotoxic proteins for further exploration on the exact mechanism involved in their cytotoxic activity against breast cancer cells with the aim of drug discovery.

##  Supplemental Information

10.7717/peerj.9650/supp-1 Supplemental Information 1 Sephadex G-50 fractionation of Ligno TG-K CWE A total of 150 fractions (2 mL of fraction/tube) were collected. The carbohydrate content was determined by phenol sulfuric method (absorbance at 490 nm) and protein content was determined by Bradford protein assay (absorbance at 595 nm). Using the protein calibration standards, the molecular weights of the HMW, MMW and LMW were estimated between ¿15 kDa, 5.0-14.0 kDa and ¡ 4.4 kDa, respectively. Abbreviations: HMW, high molecular weight; MMW, medium molecular weight; LMW, low molecular weight.

10.7717/peerj.9650/supp-2 Supplemental Information 2 Morphological changes of the MCF7 apoptotic cells Cell shrinkage, membrane blebbing (MB), nuclear compaction (NC) and apoptotic bodies (AB) were clearly observed in the HMWp (B) treated cells compared to the untreated control (A).

10.7717/peerj.9650/supp-3 Supplemental Information 3 List of Ligno TG-K HMWp proteins Ligno TG-K HMWp proteins identified by LC-MS/MS using L. rhinocerus TM02 genome as search database.

10.7717/peerj.9650/supp-4 Supplemental Information 4 Raw data

## Additional Information and Declarations

### Data Availability

The following information was supplied regarding data availability: Sephadex G-50 fractionation of Ligno TG-K cold water extract, morphological changes of the MCF7 apoptotic cells, list of proteins identified in HMWp, body weight and tumor volumes of animals and raw Western Blots uncut are available in the Supplemental Files.
