# Supplementary figures and images for: Proteins from Lignosus tigris with selective apoptotic cytotoxicity towards MCF7 cell line and suppresses MCF7-xenograft tumor growth
Source: PeerJ. 2020 Aug 4;8:e9650. doi: 10.7717/peerj.9650 (PMC7413093; doi:10.7717/peerj.9650)

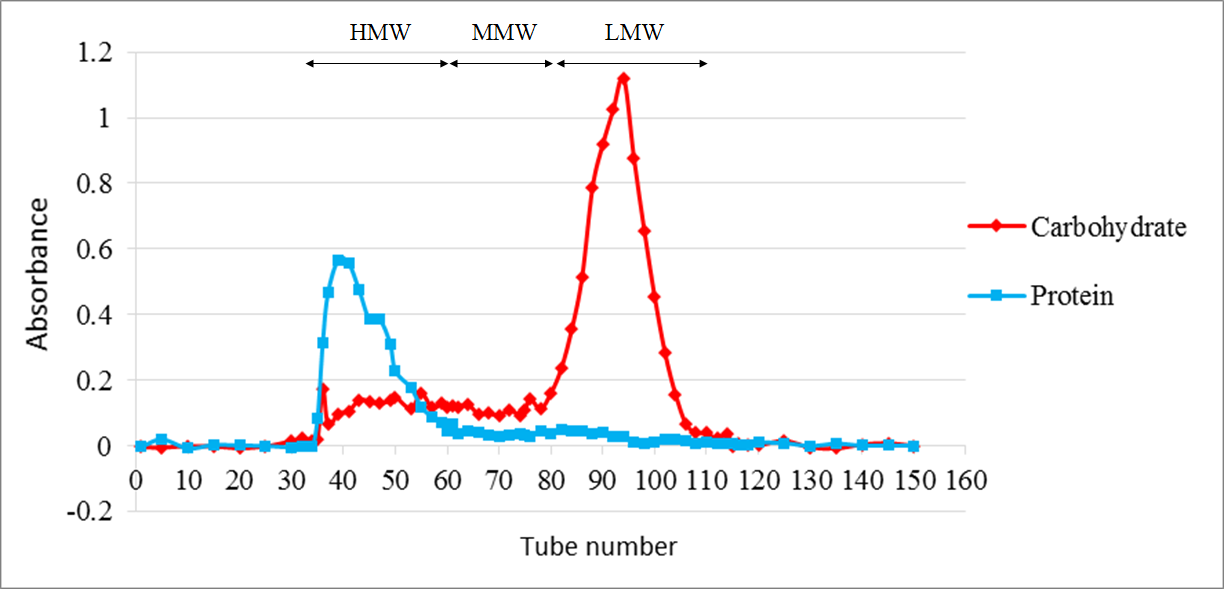

Supplement: Supplemental Information 1 — A total of 150 fractions (2 mL of fraction/tube) were collected. The carbohydrate content was determined by phenol sulfuric method (absorbance at 490 nm) and protein content was determined by Bradford protein assay (absorbance at 595 nm). Using the protein calibration standards, the molecular weights of the HMW, MMW and LMW were estimated between ¿15 kDa, 5.0-14.0 kDa and ¡ 4.4 kDa, respectively. Abbreviations: HMW, high molecular weight; MMW, medium molecular weight; LMW, low molecular weight. [file peerj-08-9650-s001.png]

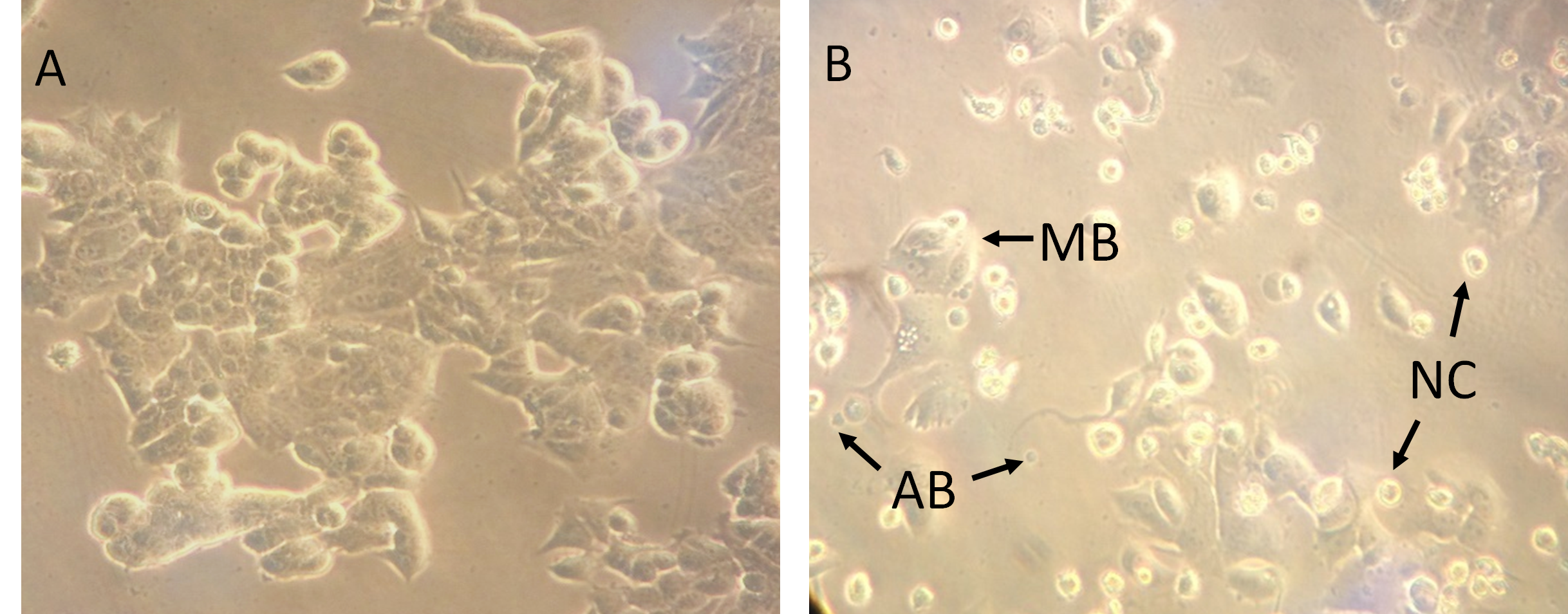

Supplement: Supplemental Information 2 — Cell shrinkage, membrane blebbing (MB), nuclear compaction (NC) and apoptotic bodies (AB) were clearly observed in the HMWp (B) treated cells compared to the untreated control (A). [file peerj-08-9650-s002.png]

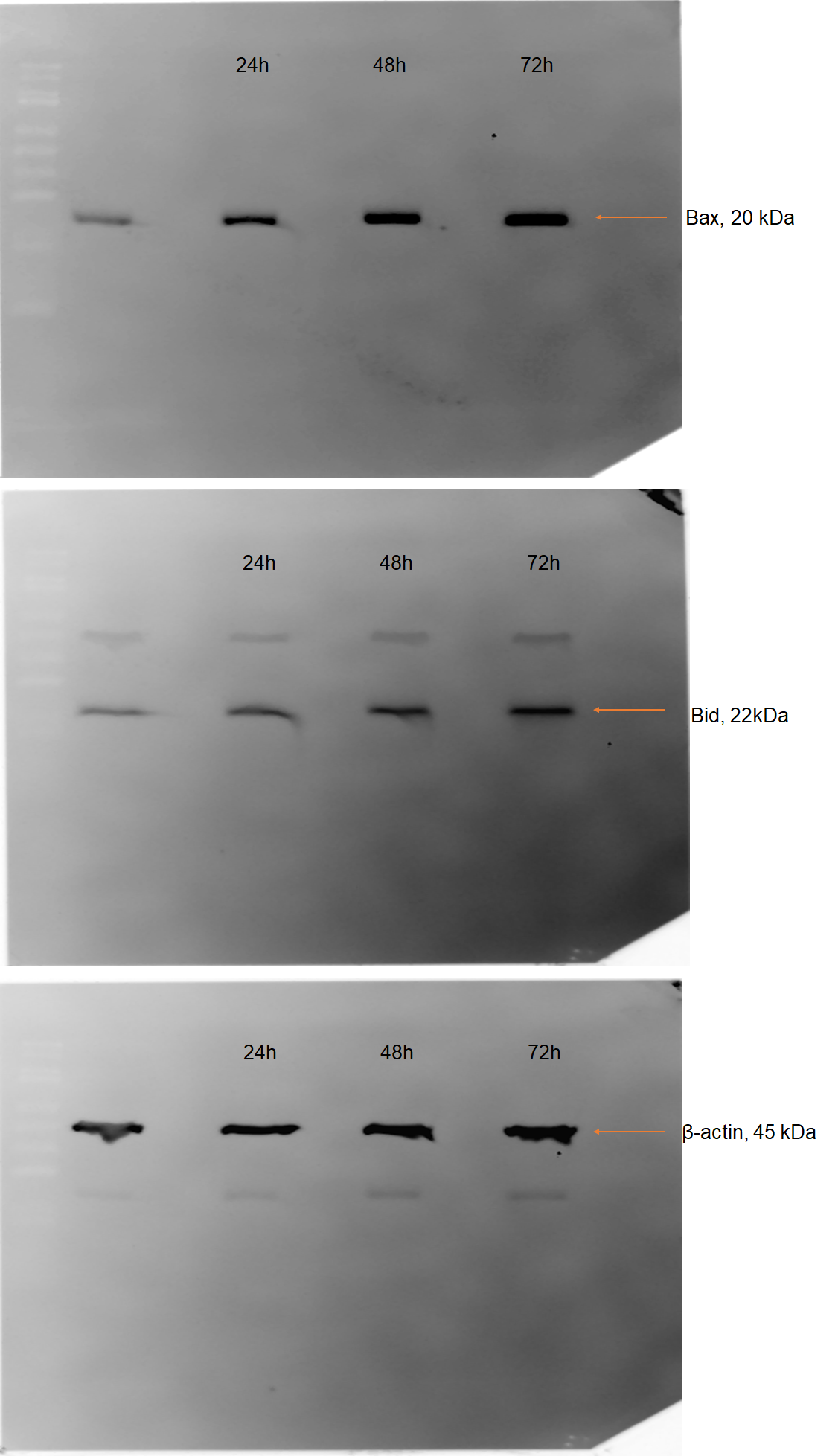

Supplement: Supplemental Information 4 [file peerj-08-9650-s004.zip › Supplemental File/HMWp-treated Bax, Bid and B-actin.png]

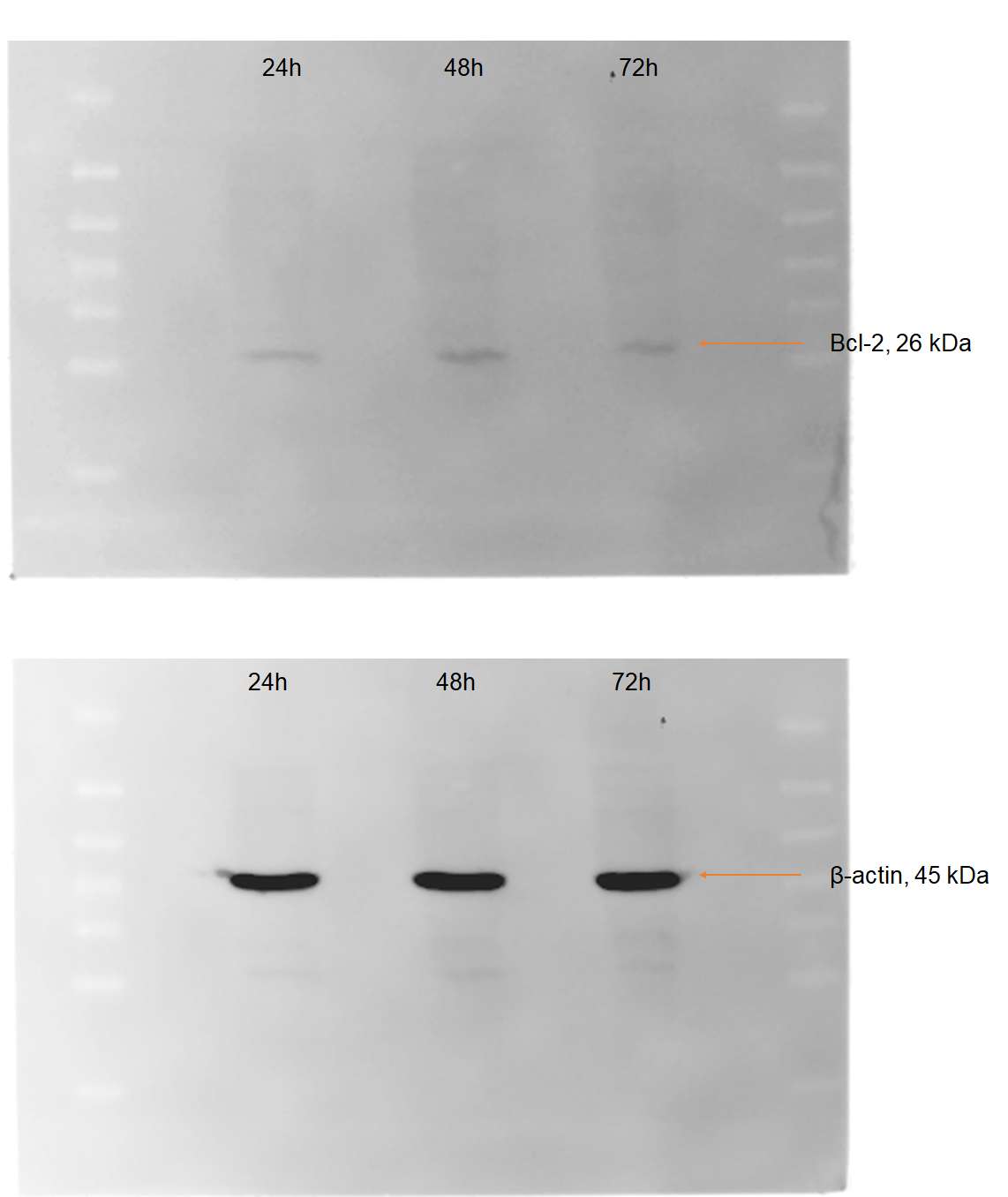

Supplement: Supplemental Information 4 [file peerj-08-9650-s004.zip › Supplemental File/HMWp-treated Bcl2 and B-actin.png]

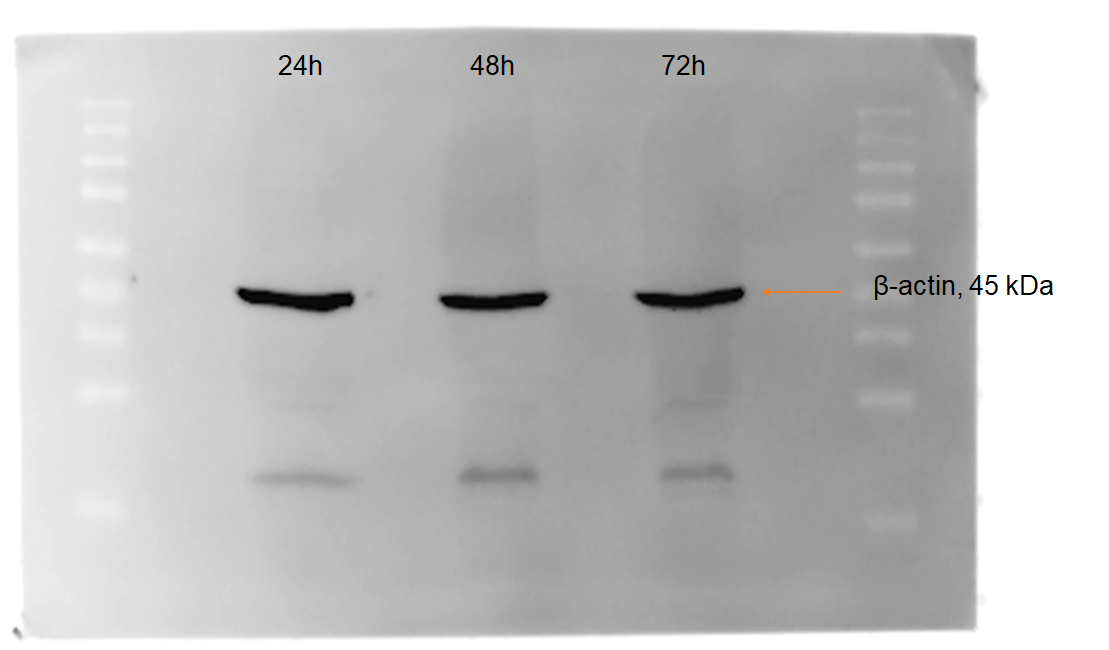

Supplement: Supplemental Information 4 [file peerj-08-9650-s004.zip › Supplemental File/Untreated B-actin.png]

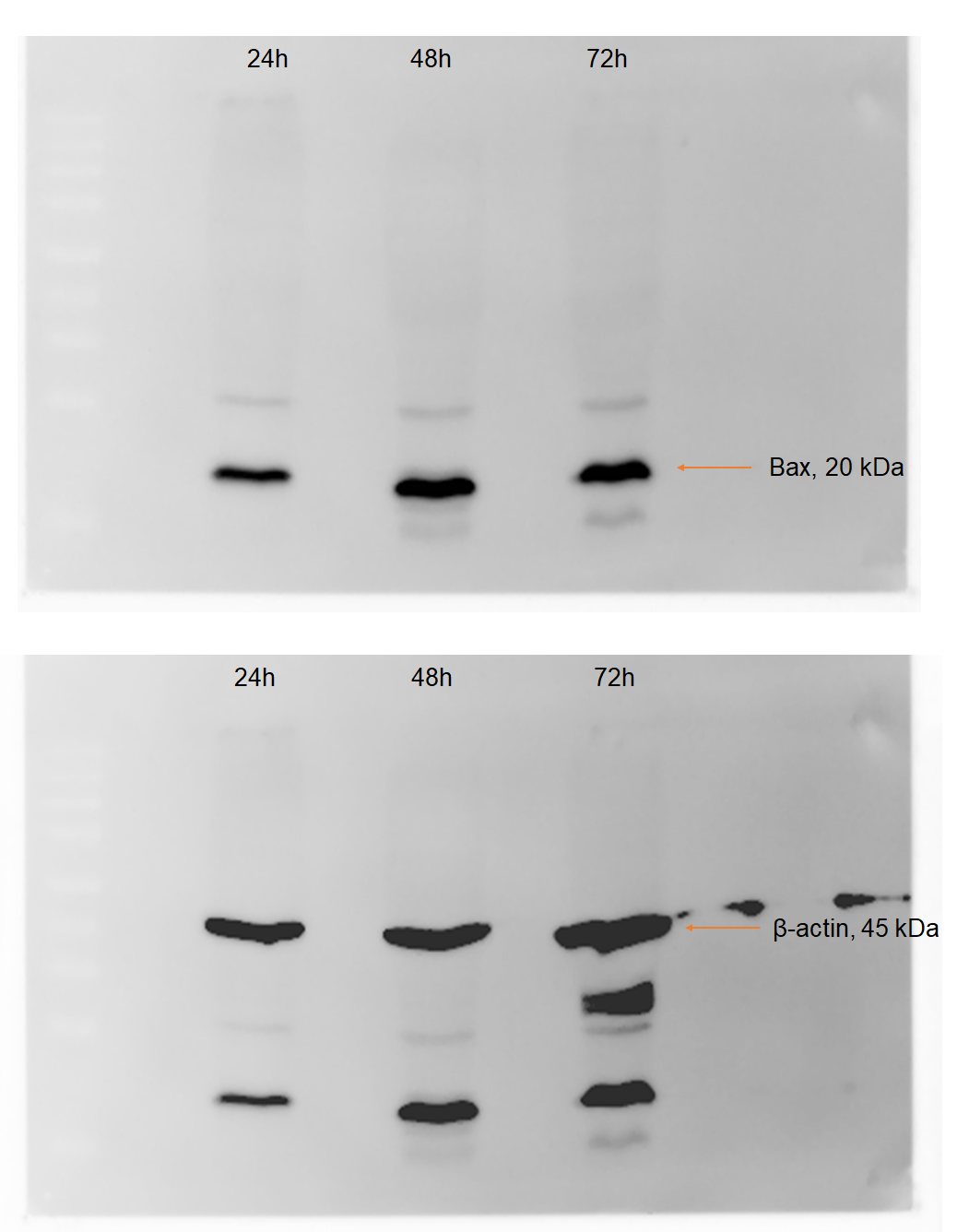

Supplement: Supplemental Information 4 [file peerj-08-9650-s004.zip › Supplemental File/Untreated Bax and B-actin.png]

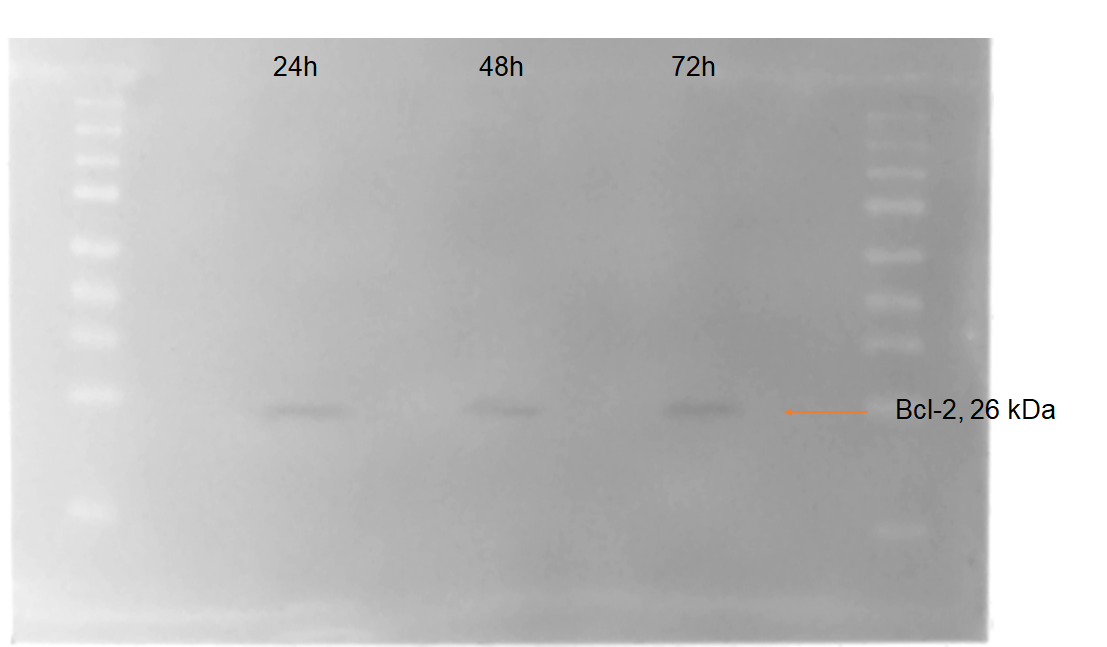

Supplement: Supplemental Information 4 [file peerj-08-9650-s004.zip › Supplemental File/Untreated Bcl-2.png]

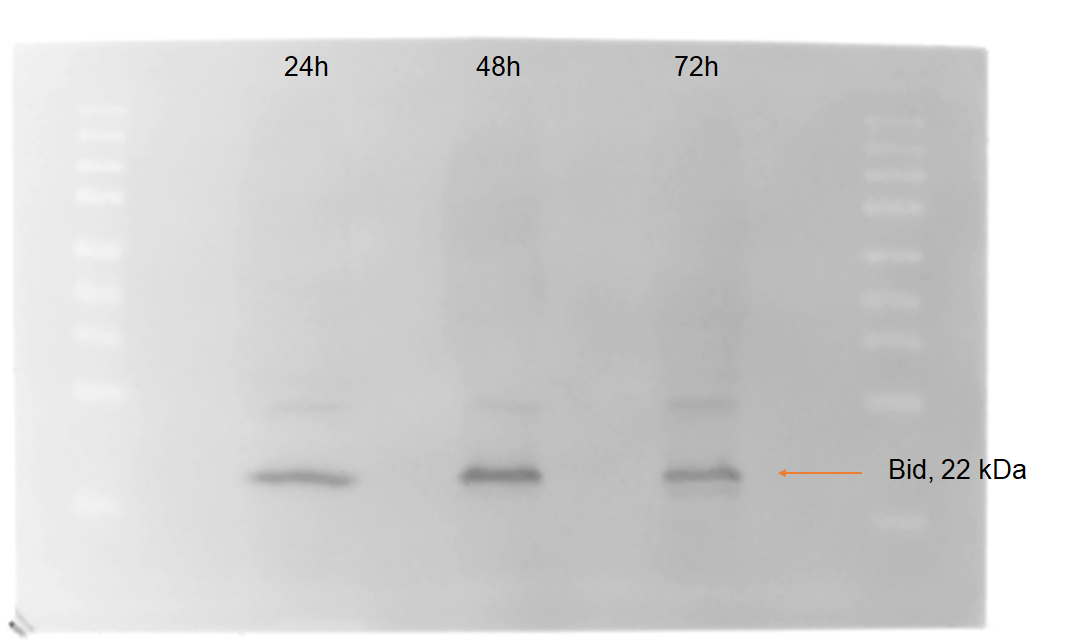

Supplement: Supplemental Information 4 [file peerj-08-9650-s004.zip › Supplemental File/Untreated Bid.png]
