# Supplementary material for: Proteins from Lignosus tigris with selective apoptotic cytotoxicity towards MCF7 cell line and suppresses MCF7-xenograft tumor growth
Source: PeerJ. 2020 Aug 4;8:e9650. doi: 10.7717/peerj.9650 (PMC7413093; doi:10.7717/peerj.9650)
Supplement: Supplemental Information 3 — Ligno TG-K HMWp proteins identified by LC-MS/MS using L. rhinocerus TM02 genome as search database. [file peerj-08-9650-s003.pdf]

Table S1:
List of Ligno TG-K HMWp proteins identified by LC-MS/MS using *L. rhinocerus* TM02 genome as search database.

Section	Spectra (#)	Distinct Peptides (#)	Distinct Summed MS/MS Search Score	% AA Coverag e	Protein MW (Da)	Protein (pI)	Mean Peptide Spectral Intensity	Protein Relative %	Database Accession #	Protein Name
S1	4	3	51.70	10.5	42840.1	5.38	9.75E+05	1.30	GME1230_g	230NA
S1	5	5	68.26	14.6	46919.6	4.86	3.00E+05	0.40	GME8314_g	Alpha-galactosidase
S1	4	4	66.10	8.2	87418.5	6.39	3.93E+05	0.52	GME2056_g	Exo-beta-1,3-glucanase
S1	4	3	48.29	15.0	19144.0	5.23	6.05E+05	0.81	GME272_g	Lectin
S1	2	2	34.37	4.9	48016.9	4.93	2.93E+05	0.39	GME4019_g	Phosphatidylserine decarboxylase-related
S1	6	3	39.84	8.0	45492.5	5.73	5.32E+06	7.08	GME4347_g	Serine protease
S2	4	2	41.25	8.5	42840.1	5.38	2.44E+06	1.04	GME1230_g	230NA
S2	3	3	50.53	10.4	39970.8	5.57	5.50E+05	0.23	GME335_g	Chain A, 3-D structure of a novel pore-forming lectin
S2	16	12	216.61	31.8	46919.6	4.86	1.76E+06	0.75	GME8314_g	Alpha-galactosidase
S2	2	2	33.29	2.4	82123.2	5.07	1.70E+06	0.72	GME7894_g	Beta-glucosidase
S2	2	2	26.57	1.9	119603.8	5.76	3.68E+05	0.16	GME8214_g	Carotenoid ester lipase precursor
S2	12	8	128.72	13.8	87418.5	6.39	5.82E+05	0.25	GME2056_g	Exo-beta-1,3-glucanase
S2	2	2	29.47	1.9	175173.6	6.71	3.43E+05	0.15	GME9535_g	Glycoside hydrolase family 79 protein
S2	5	5	73.67	6.4	90964.3	5.55	2.04E+05	0.09	GME7001_g	Glycoside hydrolase family 95 protein
S2	2	2	29.67	3.0	60459.0	5.97	3.10E+05	0.13	GME7180_g	Glyoxal oxidase precursor
S2	3	2	26.20	2.2	149581.5	6.45	4.20E+05	0.18	GME8409_g	Hypothetical protein MGG_05540
S2	3	2	36.21	3.5	54630.2	4.6	2.80E+06	1.19	GME4362_g	Hypothetical protein SCHCODRAFT_50312
S2	4	3	52.55	15.0	19144.0	5.23	2.12E+06	0.90	GME272_g	Lectin
S2	3	3	45.66	6.2	70156.9	5.75	3.70E+05	0.16	GME1854_g	Pc20g15160
S2	3	2	28.01	4.9	48016.9	4.93	4.00E+05	0.17	GME4019_g	Phosphatidylserine decarboxylase-related
S2	8	7	132.51	19.2	53784.7	6.56	1.06E+06	0.45	GME590_g	Predicted protein
S2	5	5	75.21	9.7	58776.8	5.57	2.18E+05	0.09	GME877_g	Predicted protein
S2	5	4	72.73	13.1	56897.8	4.37	9.90E+05	0.42	GME9508_g	Predicted protein

Table S1, continued

Section	Spectra (#)	Distinct Peptides (#)	Distinct Summed MS/MS Search Score	% AA Coverage	Protein MW (Da)	Protein (pI)	Mean Peptide Spectral Intensity	Protein Relative %	Database Accession #	Protein Name
S2	2	2	27.77	4.3	45492.5	5.73	5.20E+05	0.22	GME4347_g	Serine protease
S2	2	2	35.67	3.7	101472.6	6.57	2.05E+05	0.09	GME3952_g	Vacuolar protein sorting-associated protein 70
S3	4	2	45.32	8.5	42840.1	5.38	5.65E+06	2.40	GME1230_g	230NA
S3	8	5	93.82	12.6	39970.8	5.57	2.18E+06	0.93	GME335_g	Chain A, 3-D structure of a novel pore-forming lectin
S3	2	2	29.77	19.3	13281.2	9.48	2.57E+05	0.11	GME1876_g	876NA
S3	2	2	37.79	9.2	34079.5	6.92	1.10E+06	0.47	GME6852_g	Acid phosphatase
S3	4	4	73.17	11	46919.6	4.86	1.19E+05	0.05	GME8314_g	Alpha-galactosidase
S3	4	4	56.66	6.7	60026	5.45	2.14E+05	0.09	GME5195_g	ATP-dependent Zn protease
S3	3	3	41.11	4	82123.2	5.07	2.27E+05	0.10	GME7894_g	Beta-glucosidase
S3	2	2	25.34	5.3	56697	6.46	1.16E+05	0.05	GME5047_g	Bilirubin oxidase
S3	2	2	26.07	6.7	54002.5	5.46	6.35E+04	0.03	GME1840_g	Cellobiohydrolaseii
S3	6	5	94.05	7.6	87418.5	6.39	3.10E+05	0.13	GME2056_g	Exo-beta-1,3-glucanase
S3	7	6	81.69	4.6	149581.5	6.45	8.86E+05	0.38	GME8409_g	Hypothetical protein MGG_05540
S3	2	2	30.33	7.9	36148.2	5.21	1.86E+05	0.08	GME6304_g	Hypothetical protein NECHADRAFT_82658
S3	2	2	33.11	3.3	56823.1	4.84	6.20E+05	0.26	GME4454_g	Hypothetical protein SCHCODRAFT_258939
S3	3	2	34	3	74862.7	5.48	3.03E+06	1.29	GME2803_g	Hypothetical protein SCHCODRAFT_66537
S3	12	9	157.19	16.4	64157.9	5.21	1.32E+06	0.56	GME8954_g	Hypothetical protein SCHCODRAFT_78645
S3	10	7	120.58	10.4	76535.8	6.74	9.61E+05	0.41	GME3033_g	Hypothetical protein SCHCODRAFT_78645
S3	5	4	69.75	19.6	19144	5.23	2.28E+06	0.97	GME272_g	Lectin
S3	2	2	25.16	6.8	29562.9	6.93	2.22E+05	0.09	GME271_g	Lectin
S3	2	2	24.07	3.3	70277.1	5.55	1.13E+05	0.05	GME1357_g	Mannose-6-phosphatase
S3	3	3	52.72	10	41772.9	5.25	4.67E+05	0.20	GME3687_g	Polygalacturonase
S3	3	2	36.95	5.4	56897.8	4.37	1.66E+05	0.07	GME9508_g	Predicted protein

Table S1, continued

Section	Spectra (#)	Distinct Peptides (#)	Distinct Summed MS/MS Search Score	% AA Coverage	Protein MW (Da)	Protein (pI)	Mean Peptide Spectral Intensity	Protein Relative %	Database Accession #	Protein Name
S3	2	2	27.77	5.4	34997.3	6.26	1.45E+05	0.06	GME4952_g	Predicted protein
S3	3	2	34.3	5.9	45492.5	5.73	1.80E+06	0.76	GME4347_g	Serine protease
S3	2	2	31.26	5.9	41051.9	6.58	1.57E+06	0.67	GME5911_g	Serine protease
S4	6	3	67.01	10.3	42840.1	5.38	1.66E+06	7.61	GME1230_g	230NA
S4	2	2	38.42	7.8	48560.2	5.51	6.25E+05	2.86	GME10601_g	Agmatinase
S4	4	3	51.35	17.4	21871.2	5.25	1.10E+06	5.02	GME6918_g	Hypothetical protein BC1G_08630
S4	4	2	30.68	4.1	64157.9	5.21	2.02E+05	0.92	GME8954_g	Hypothetical protein SCHCODRAFT_78645
S4	7	5	93.82	43.9	19144	5.23	9.11E+05	4.17	GME272_g	Lectin
S4	2	2	30.2	6.8	29562.9	6.93	2.86E+05	1.31	GME271_g	Lectin
S4	5	5	72.16	19.4	34997.3	6.26	2.70E+05	1.24	GME4952_g	Predicted protein
S4	2	2	35.66	5.4	56897.8	4.37	4.13E+05	1.89	GME9508_g	Predicted protein
S4	3	2	30.64	10.5	32639.9	5.7	2.48E+05	1.13	GME3927_g	Predicted protein
S4	3	2	35.99	8.2	39857.5	5.86	1.26E+06	5.75	GME3216_g	Rnase Gf29
S4	3	2	35.39	4.4	89414.7	5.83	4.53E+05	2.07	GME7804_g	Serine protease
S5	4	2	45.01	8.5	42840.1	5.38	2.03E+06	7.73	GME1230_g	230NA
S5	4	3	44.83	20.5	16740.1	7.98	3.63E+05	1.38	GME7698_g	Hypothetical protein SCHCODRAFT_74436
S5	10	7	117.22	53.1	19144	5.23	2.01E+06	7.66	GME272_g	Lectin
S5	2	2	29.73	15.8	16747.6	5.27	7.65E+04	0.29	GME2200_g	Lectin
S5	2	2	22.91	5	36782.1	5.78	1.27E+05	0.48	GME10160_g	Thioredoxin reductase
S6	4	3	53.94	12.1	42840.1	5.38	2.95E+05	1.76	GME1230_g	230NA
S6	3	2	31.83	19.3	13281.2	9.48	7.87E+05	4.68	GME1876_g	876NA
S6	3	2	36.55	6.4	38313.2	4.88	3.29E+05	1.96	GME9837_g	Hypothetical protein SCHCODRAFT_103662
S6	4	2	29.96	13.9	16740.1	7.98	2.20E+05	1.31	GME7698_g	Hypothetical protein SCHCODRAFT_74436

Table S1, continued

Section	Spectra (#)	Distinct Peptides (#)	Distinct Summed MS/MS Search Score	% AA Coverage	Protein MW (Da)	Protein (pI)	Mean Peptide Spectral Intensity	Protein Relative %	Database Accession #	Protein Name
S6	5	4	62.86	19.6	19144	5.23	2.28E+05	1.36	GME272_g	Lectin
S6	3	2	27.12	16.6	18104.5	7.99	1.32E+06	7.85	GME4605_g	Predicted protein
S6	7	4	53.81	15.3	36782.1	5.78	2.57E+05	1.53	GME10160_g	Thioredoxin reductase
